# Analysis of a Process for Producing Battery Grade Lithium Hydroxide by Membrane Electrodialysis

**DOI:** 10.3390/membranes10090198

**Published:** 2020-08-25

**Authors:** Mario Grageda, Alonso Gonzalez, Adrian Quispe, Svetlana Ushak

**Affiliations:** Departamento de Ingeniería Química y Procesos de Minerales and Center for Advanced Study of Lithium and Industrial Minerals (CELiMIN), Universidad de Antofagasta, Campus Coloso, Av Universidad de Antofagasta, 02800 Antofagasta, Chile; alonso.gonzalez@celimin.com (A.G.); adrian.quispe.huayta@ua.cl (A.Q.); svetlana.ushak@uantof.cl (S.U.)

**Keywords:** lithium hydroxide, membrane electrodialysis, lithium brines, operating conditions, electrochemical kinetic, specific electrical consumption

## Abstract

A membrane electrodialysis process was tested for obtaining battery grade lithium hydroxide from lithium brines. Currently, in the conventional procedure, a brine with Li+ 4–6 wt% is fed to a process to form lithium carbonate and further used to produce lithium hydroxide. The disadvantages of this process are its high cost due to several stage requirement and the usage of lime, causing waste generation. The main objective of this work is to demonstrate the feasibility of obtaining battery grade lithium hydroxide monohydrate, avoiding production of lithium carbonate. A laboratory cell was constructed to study electrochemical kinetics and determine energetic parameters. The effects of current density, electrode material, electrolyte concentration, temperature and cationic membrane (Nafion 115 and Nafion 117) on cell performance were determined. Tests showed that a current density of 1200 A/m^2^ and temperatures between 75–85 °C allow reduced specific electricity consumption (SEC) (7.25 kWh/kg LiOH). A high purity product is obtained at temperatures below 75 °C, with a Nafion 117 membrane and low electrolyte concentration. Resulting key electrochemical data would enable a pilot-scale process implementation to obtain lithium compounds.

## 1. Introduction

Lithium possesses unique properties, making this material the most promising for electrical and thermal energy storage applications [1,2]. Its application in lithium batteries enables power sources with high energy density, particularly in applications where mass is a considerable factor. Lithium batteries have large energy charge and discharge capacity and no memory effect. They are suitable for application in mobile devices, electric vehicles and energy storage systems. In batteries, lithium is used for manufacturing cathodic materials: LiCoO_2_, LiFePO_4_, Li_2_MnO_4_, among others. For this purpose, lithium salts such as lithium carbonate and lithium hydroxide are used as raw materials, requiring high purity for their application. Battery grade lithium hydroxide monohydrate is a high purity product (min 99.3%). It is suitable for application in the production of cathodic materials for lithium ion batteries and is used as raw material for the production of other high purity lithium compounds.

Currently, the process to obtain lithium hydroxide begins with lithium brine concentrated in evaporation ponds with a lithium content of 4.0 to 6.0 wt% [3]; being equivalent to 25–40 wt% of LiCl. This brine is then purified in one or two stages to remove impurities, mainly residual magnesium and calcium. The purified brine, previously filtered to separate suspended solids, is treated with sodium carbonate at 90–95 °C to precipitate the lithium as lithium carbonate. Subsequently, to obtain lithium hydroxide a reaction of lithium carbonate with lime is used [4,5]. Although this process is not complex, it has several disadvantages. For example, to obtain high quality LiOH∙H_2_O, a high degree of purity of the starting components is required. Moreover, taking into account the low solubility of Ca(OH)_2_, this process is characterized by LiOH low concentration in solution and great Li^+^ loss dragged by calcium carbonate as solid waste [5,6,7]. For this reason, it is necessary to develop an alternative process with less processing steps allowing for the attainment of lithium hydroxide concentrated solutions. Furthermore, the process must have lower lithium losses, lower initial cost, reduced chemical reagent consumption and is desired to be environmentally friendly.

The electro-membrane processes are modern methods for separation and concentration of various ionic species [8,9,10,11,12]. Their application has been primarily for industrial wastewater treatment, seawater desalination, and production of substances for food processing, among others. 

There is some history of experimental evaluation processes using nanofiltration membranes for lithium chloride recovery from brines containing high concentrations of lithium and magnesium, boron and sulfate [13]. However, the results show that lithium recovery using this type of membrane has economically poor behavior compared to electro-membrane processes. In recent years, several studies have been conducted relating electro-membrane processes and lithium [14,15,16,17,18,19]. Parsa et al. [14] studied an electrodialysis process for the recovery of lithium from sodium-contaminated lithium bromide solution, the effects of voltage and concentration were observed. Some studies focused on electrodialysis for separation of Li^+^/Mg^2+^ from salt-lake brines according to different voltage operating conditions, linear velocities, feed and special selective membranes or electrodes [15,16,17]. Zhou et al. [18] worked on the concentration of a lithium sulfate solution by electrodialysis. In their work, the effects of membrane type, voltage, and operating mode were investigated, obtaining effective lithium extraction. Other recent studies considered bipolar membranes for lithium recovery and lithium hydroxide production [20,21,22,23,24]. Jiang et al. [22] presented interesting results in obtaining LiOH from Lake Brines using bipolar membrane electrodialysis. In their work, they used aqueous Li_2_CO_3_ solutions between 0.054 M and 0.18 M obtaining current efficiencies between 91.8–94.2% and an energy consumption of 6.66 kWh/kg LiOH at 300 A/m^2^. Melnikov et al. [23] used bipolar membrane electrodialysis to treat a LiCl solution with 1.8–59% organic solvents and produce up to 0.3M LiOH, achieving a current efficiency of 60% and an energy consumption of 6.6 kWh/kg. On the other hand, Bunani et al. [24], by means of bipolar membrane electrodialysis, studied simultaneous recovery of lithium and boron from aqueous solutions at different applied voltages, reporting recovery efficiencies higher than 90%. They indicated that the current efficiency decreases with applied voltage.

For lithium recovery using membrane-based technologies, Li et al. [12] mentioned that these processes are feasible but are constrained by high capital and operating costs. In addition, there was the difficulty that at high concentrations other ions interfere with the process. Chen et al. [25], studied the effect of coexisting cations in a lithium recovery process by selective-electrodialysis. Higher competitiveness was observed with monovalent cations due to their lower hydration ratio, and lithium recovery was higher at lower concentrations of other cations. On the other hand, Cassady et al. [26] compared the permselectivity for cationic membranes with different degrees of functionalization and in contact with different salts. They reported that membrane permselectivity for LiCl was higher compared to other salts. 

Regarding selective lithium extraction, an interesting work was carried out by Liu et al. [17], using membrane electrolysis with LiFePO_4_ electrodes selectively extracting lithium from LiCl brine. In their work they determined an optimal electrode spacing of 2 cm. Recently, Razmjou et al. [27] studied the application of sub-nanometer hydrous phyllosilicate channels on membranes for the selective extraction of lithium, excluding other anions and divalent cations of lower ionic mobility than lithium.

Some patents exist concerning lab-scale studies of lithium hydroxide concentration and production from brines by electro-membrane processing [28,29,30]. Brown [28], Harrison and Blanchet [29] and Buckley et al. [30] describe a process in which a brine containing lithium is concentrated by membrane electrolysis to form LiOH. In the electrolytic cell, a cationic membrane separates anolyte from catholyte, and lithium ions migrate through the membrane to form aqueous lithium hydroxide in the catholyte. Currently, to obtain sodium and potassium hydroxide, electro-membrane processes are used among other technologies [31,32], where the use of ion exchange membranes proves to be ideal for cations capture. The most commonly used membranes in alkaline processes are Nafion by DuPont, which exhibit the property of blocking the hydroxyl ions. This option has wide industrial application in the chlor-alkali process and in fuel cells [33]. 

As described above, several studies are underway for the recovery of lithium using membrane-based technologies. However, none of these technologies have reached industrial application and most of the focus is on membrane application at low electrolyte concentrations. The objective of this work is to study the performance of an electrodialysis membrane-based process to obtain lithium hydroxide monohydrated adequate for brines from Salar de Atacama (Chile) and determine process operating parameters. Thus, the objective is to generate knowledge and foundations to continue using this technique in obtaining a high purity product and subsequently implement a pilot-scale process. 

## 2. Materials and Methods 

### 2.1. Reactives and Materials

The raw materials used were panreac quality, this means of a purity equal to or greater than 99%, of the product line Merck (Darmstadt, Germany). Deionized water with a 0.054 mS/cm conductivity was used to prepare solutions. Five separate solutions, two anolytes and three catholytes were used. As anolyte, synthetic LiCl solutions with traces of impurities were used as shown in Table 1. The chemical composition of the anolyte is based in our previous research [34], where a concentrated lithium brine was purified in order to obtain magnesium concentration below 0.001 wt%. Anolyte 1 and anolyte 2 simulate a purified lithium brine in different dilution conditions, approximately 14 and 32 wt% LiCl, respectively. As a catholyte, three different LiOH solutions with concentrations of 1.15, 2.30 and 5.70 wt% were used. 

### 2.2. Cell Design and Membrane Electrodialysis System

**Figure 1:**
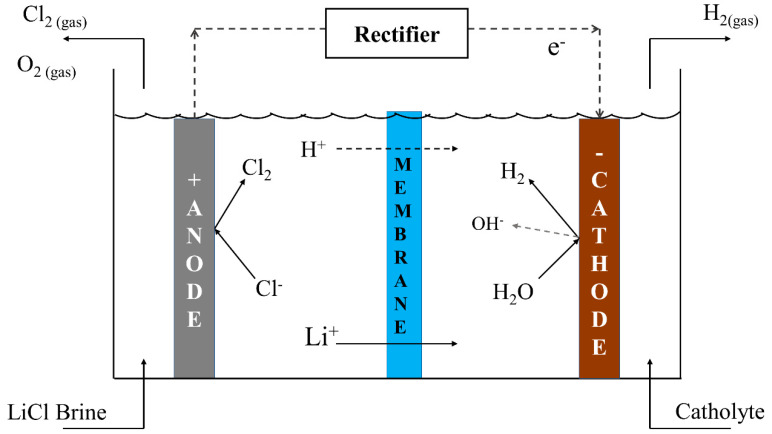
Diagram of membrane electrodialysis cell to obtain Lithium hydroxide.

This work involves the usage of membrane electrodialysis as a technique for separation of lithium ions and lithium hydroxide production. Figure 1 shows the method for obtaining LiOH in a membrane electrodialysis cell; a Li^+^ ion is transferred through a cationic membrane into a cathode compartment. In the cathodic half-reaction, an OH^−^ ion is generated and LiOH is produced. 

The overall system chemical reaction for obtaining lithium hydroxide is defined as follows:


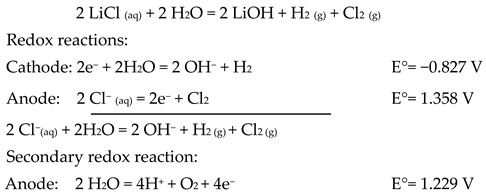


**Figure 2:**
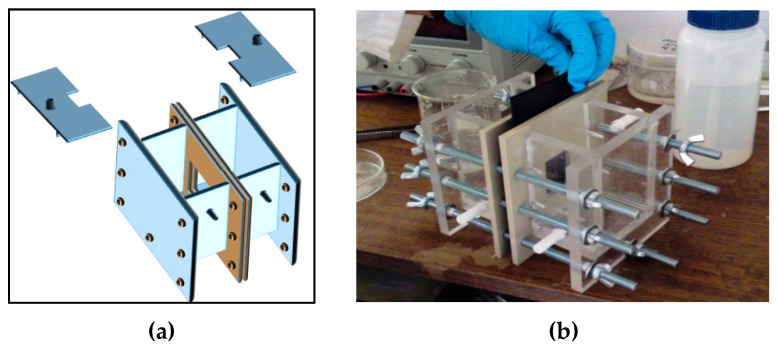
Membrane electrodialysis cell design with two compartments. (**a**) Computer design, (**b**) cell built in acrylic.

The acrylic cell used in the experiment is made up of two compartments with simple rectangular geometry (Figure 2), a first one containing cathode and catholyte, a second one containing anode and anolyte. In addition, both compartments have inlet and outlet holes for recirculation and stirring. The cathode and anode compartments are separated by a cationic membrane in order to allow transport of lithium ions. The membrane was fitted in a 4 cm × 3 cm acrylic window between compartments. To avoid electrolyte leakage, the membrane was placed between 2 mm thick rubber seals. The acrylic cell was not a sealed model, so a fume hood was used for gas extraction. On the other hand, output flow from the cell corresponds to a gravity flow, so that linear flow in the catholyte and anolyte compartments was limited to approximately 0.2–0.3 cm/s.

Nafion 115 and Nafion 117 membranes (DuPont Co., Wilmington, DE, USA) were used, with an effective area of 12 cm^2^. These membranes differ on the number of repeating monomer units and thickness, which were 0.13 and 0.18 mm, respectively. Membranes were pre-conditioned by an immersion technique phase in a solution of LiOH 2.30 wt% for 72 h at room temperature. Before each experiment, membranes were washed with distilled water.

Three types of electrodes were used: a stainless steel 316 sheet and a nickel square rod 99.9% (Votorantim Metais Niquel S.A, Fortaleza de Minas, Brazil) as the cathode (with an exposed area of 12 cm^2^) and a graphite rod 99.9% (Brunssen, Guadalajara, Mexico) as the anode with an exposed area of 24 cm^2^. To remove the surface layer of oxide, electrodes were submerged in a solution of HNO_3_ at 10 wt% during 30 min. The cathode–anode distance was 20 mm. The use of different electrode geometries and materials was not part of this study, however, electrode geometry and its influence on current distribution lines and electrochemical kinetics must be taken into account for an optimal larger scale cell design. Use of electrodes of different geometry can cause a decrease in current efficiency. Such inhomogeneity can be compensated by reducing the distance between electrodes and using electrolytes with high conductivity, as is the case of electrolytes in this work.

**Figure 3:**
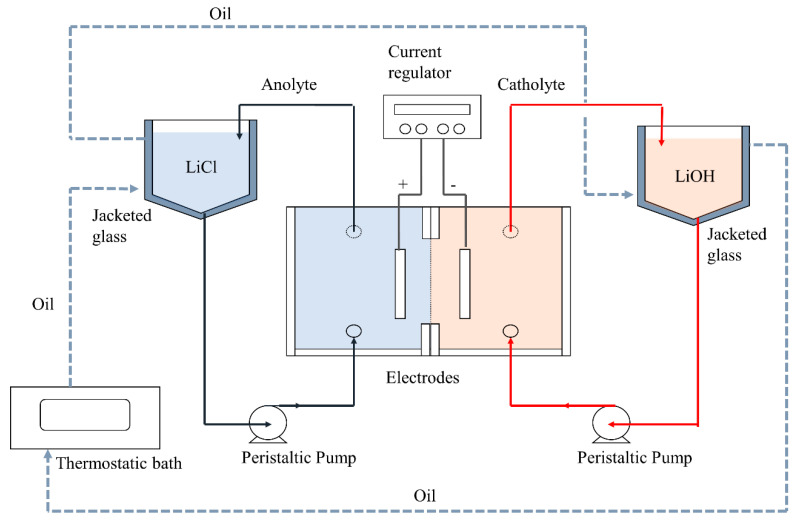
Schematic diagram of lab scale process to obtain LiOH by membrane electrodialysis.

Figure 3 shows the experimental scheme of the batch process at laboratory scale. Electrolytes were recirculated with a constant flow across the cell using peristaltic pumps (Watson-Marlow 520SN/R2, Falmouth, UK). Electric current is provided using a rectifier (GW Instek GPR-1810HD, New Taipei, Taiwan). 

The total volume for anolyte and catholyte were 700 cm^3^ each. Electrolytes temperatures were kept constant by a Julabo thermostatic bath (Seelbach, Germany), injected through double-jacket recirculation tanks. A pH-meter Model 50 (Denver Instrument Company, Arvada, Colorado, CO, USA) was used to measure electrolyte pH during the experiment. 

Theoretical mass transfer of lithium ions (*m_theor_*) through the membrane is determined by Faraday’s laws of electrolysis (Equation (1)):

(1) $$m_{theor}\;=\frac{I\cdot t\cdot M}{z\cdot F}$$

where *I* is current intensity (A), *M* is molecular weight (g/mol), z is the number of electrons per ion, *F* is Faraday’s constant (96484.5 As/mol) and *t* is the time interval (s).

The current efficiency (Ø) was calculated by Equation (2):

(2) $$Ø\;=\frac{z\cdot F}{I\cdot t}\Delta N_{i}$$

where *∆N_i_* is the mol difference of specie “*i*” between the final and initial catholyte, *I* is current intensity (A), *z* is the valence number, *F* is Faraday’s constant (96484.5 As/mol) and *t* is time.

Specific electrical consumption (*SEC*) is calculated by Equation (3):

(3) $$SEC\;=\;\frac{V_{cell}\cdot I\cdot t}{m_{exp}}$$

where *V_cell_* is cell voltage, *I* is current intensity and *m_exp_* is the experimental mass of LiOH produced by membrane electrodialysis processing.

### 2.3. Evaporation and Crystallization

In order to obtain LiOH∙H_2_O crystals, after each experiment evaporation and crystallization of the final catholyte was conducted by rotavapor Buchi R-210 (Buchi Corp., New Castle, De, USA), forming crystals by vacuum evaporation effect. Evaporation and crystallization were performed at 24.10 mmHg pressure and 40 °C temperature. Subsequently, the obtained crystals were washed and dried in a CO_2_ free atmosphere.

### 2.4. Operating Conditions

Experiments were carried out in a lab-scale cell. The effects of current density, concentration, temperature, membrane type and electrode material were studied.

The trading volume for each compartment was 225 cm^3^ with a recirculation rate of 120 cm^3^/min, corresponding to an electrolytes linear flow of 0.3 cm/s. The electrodes used were nickel and stainless steel 316 as cathodes and graphite as anode, with current densities of 1200, 2400 and 3600 A/m^2^ and temperatures of 25 °C, 50 °C, 75 °C and 85 °C. The cell was operated over 4 h, taking samples from both compartments to assess evolution of chemical composition. Table 2 shows operating conditions for each experiment.

### 2.5. Potentiodynamic Sweeps 

Electrochemical kinetics of anodic and cathodic half-reactions were characterized by potentiodynamic sweeps. Tests were carried out using an Autolab PGSTAT302N potentiostat (Metrohm, Herisau, Switzerland). To study water reduction to gaseous hydrogen, the working electrodes were a bar of nickel and a sheet of stainless steel 316, the counter electrode was a graphite rod. To study water oxidation to oxygen gas and chloride oxidation to gaseous chlorine, the working electrode was graphite and the counter electrode was a nickel bar. The nickel bar, the stainless steel 316 and graphite rod had an apparent surface area of 1.00, 2.70 and 2.87 cm^2^, respectively. The reference electrode was Ag/AgCl 3 M KCl (0.194 V vs. standard hydrogen electrode (SHE)), the scan rate was 1 mV/s. LiOH as a catholyte and LiCl as anolyte were the solutions used during the experiment. Electrolyte temperatures were kept constant at 75 °C and 85 °C by a Julabo thermostatic bath, injected through double-jacket recirculation tanks. Electrolyte agitation was kept constant at 120 cm^3^/min by recirculation using Watson-Marlow 520SN/R2 peristaltic pumps. 

### 2.6. Final Product Characterization

Chemical analysis was performed on LiOH∙H_2_O crystal samples to determine chemical composition and concentration range of main impurities. Sodium, potassium, calcium, lithium and magnesium concentrations were determined by atomic absorption spectrometry. Chloride and sulfate were determined by volumetric titration with AgNO_3_ and BaCl_2_, respectively. Moisture was determined by drying until constant weight at 40 °C was achieved in an inert atmosphere.

Sample composition was analyzed by an X-ray Powder Diffractometer. Powdered sample was positioned on a flat plate sample holder after sample powdering in an agate mortar. This technique was used to characterize crystallographic structure by comparing obtained diffraction data with data from a database maintained by the International Centre for Diffraction Data (www.icdd.com). Analysis of X-ray diffraction was performed on an X-Ray diffractometer SIEMENS model D5000 (40 kV, 30 mA); radiation of Cu Ka1 (l = 1.5406 Å); vertical Bragg-Brentano; scan range: 3–70° 2q; step size: 0.020° 2q; step time: 1.0 s. 

## 3. Results

### 3.1. Cell Parameter Performance

Table 3 shows results obtained in each of the experiments. Production rate, average cell voltage, current efficiency relative to transferred Li^+^ mass, specific electrical consumption (SEC) and purity of product are presented. 

Experiments 1, 2 and 3 provide results for different current densities. Experiments 2, 4, 9 and 10 allow comparison of results at four different temperatures. In a similar way, if experiments 2 and 6 are compared and the effect of ion exchange membrane type can be observed. In addition, experiments 2, 8 and 11 show results when using three different initial catholyte concentrations. Experiments 4 and 5 and experiments 6 and 7 provide results for two different cathode types. On the other hand, experiment 12 presents resulting data for high concentration anolyte usage.

Each of the different parameters was analyzed. Their effects on parameters such as LiOH production rate, voltage, current efficiency, specific electrical consumption and purity of monohydrate lithium hydroxide were determined.

### 3.2. Cell Voltage Versus Time

**Figure 4:**
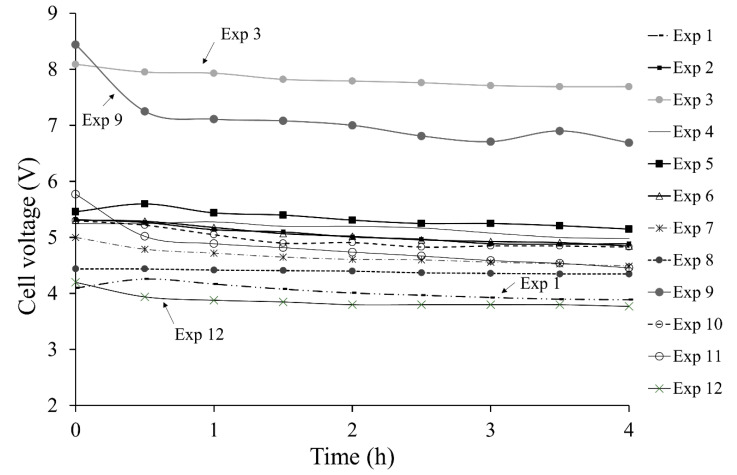
Cell voltage versus time for each experiment.

Figure 4 presents cell voltage variation over time for all experiments. Obtained cell voltage in all experiments ranged from 3.86 to 7.82 V. In all cases, a slight decrease in cell voltage occurred, which was attributed to an increase in electrolyte conductivity as related to catholyte concentration increase. After two h of operation, in most tests a relatively constant voltage was reached with an average variation of 2.5%. For each experiment, different voltages corresponding to 1200, 2400 and 3600 A/m^2^ were observed, as expressed by the relation between current density and cell voltage, and explained by Equation (4):

*V_cell_* = *ΔE_e_* + *η_a_* + |*η_c_*| + (*IR*)*_a_* + (*IR*)_*c*_ + (*IR*)_*m*_ (4)

where $\Delta E_{e}$ is the equilibrium potential; *η_a_* and *η_c_*, the anodic and cathodic overpotential; *(IR)_a_*, *(IR)_c_*, and *(IR)_m_* the anolyte, catholyte and membrane potential drops [35]. 

Lowest cell voltage was obtained in experiment 12 by using the lowest current density and the highest anolyte concentration (32 wt% LiCl), followed by experiment 1 where the lowest current density was also used. In the other hand, highest voltage was obtained in experiment 3, where the highest current density was used; also, experiment 9 with the lowest temperature shows a high cell voltage. According to Equation (3), the results show that a low cell voltage implies a specific electrical consumption (SEC) decrease. However, the LiOH production rate was also decreased.

### 3.3. PH Variation in the Membrane Electrodialysis Cell

**Figure 5:**
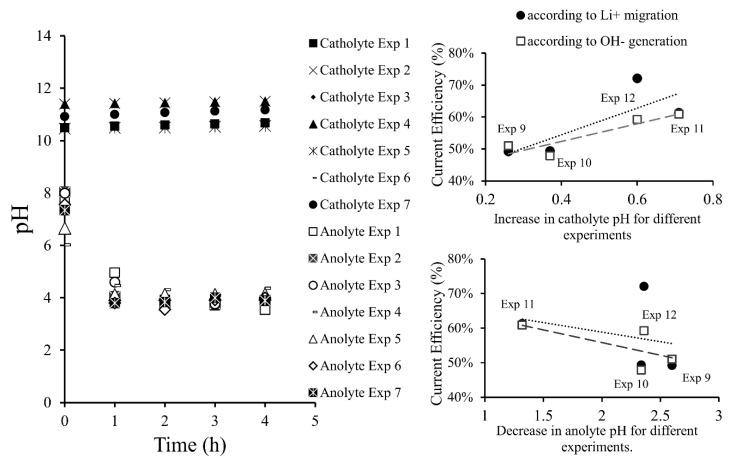
pH variation in electrolytes and its influence on current efficiency.

In Figure 5, variation of the pH over time is showed, the most representative results are presented. In the anolyte, pH decreased with time over a range between 8.05 and 3.90. This was attributed to a secondary oxidation half-reaction of water at the anode, where H^+^ was generated. After two h of operation, pH values were reduced by an average of 40%, after this period they were only reduced by approximately 1%. However, the H+ formation rate would be constant, but its values were not clearly reflected due to pH logarithmic behavior.

On the other hand, a percentage of formed protons migrated from anolyte to catholyte through the cationic membrane, a process being carried out by the Grotthuss mechanism. Transport of charge occurred by protons jumping from hydronium (H_3_O^+^) to a water molecule in chained form until reaching the membrane surface and subsequently passing through. In the catholyte, pH values were measured to be between 10.5–11.5 and exhibited an increasing trend between 1% and 7%. Although, formation of OH^−^ ions occurred at the cathode. The authors attribute this variation to a “neutralizing effect” caused by H^+^ ion migration through the membrane from the anode compartment to the cathode compartment, then H^+^ protons reacted with OH^−^ ions to generate H_2_O. This reduced the LiOH formation rate. Therefore, the catholyte pH consistently showed small change, presenting values around 10.5–11.5. 

Among the results presented in Figure 5, it can be seen that current efficiency is proportional to pH change in the catholyte and anolyte. A greater pH increase in the catholyte implied a greater rate of OH^−^ formation. On the other hand, a smaller decrease in anolyte pH implied a higher current efficiency. This was explained by the fact that less H^+^ was produced in the secondary reaction of oxygen evolution, prioritizing the oxidation semi-reaction from Cl^−^ to Cl_2_. Thus, there was more Li^+^ in the anolyte available to migrate to the catholyte. At the same time, less H^+^ migrated to the cathode avoiding the neutralization of OH^−^ and favoring the formation of LiOH in the catholyte. LiOH production efficiency depended on both Li^+^ migration and OH^−^ generation. Current efficiency with respect to lithium migration was higher compared to the one calculated according to the OH^−^ generation. This indicates that the process would be limited by unwanted migration of H^+^ protons to the cathode.

## 4. Discussion

### 4.1. Influence of Current Density

Current density effects can be observed in experiments 1, 2 and 3 (see Table 3). The results show that current density influences cell voltage, specific electrical consumption, current efficiency and LiOH production rate.

At the same temperature, an increase in current density from 1200 to 2400 A/m^2^ produced a cell voltage increase of 24%. This caused an increase in LiOH production rate of 120% at the cost of increasing specific electrical consumption by 14%. On the other hand, a current density increment from 2400 to 3600 A/m^2^ produced a 56% increase in cell voltage. This effect allows a LiOH production rate increase of 66% at the cost of increasing specific electrical consumption by 41%. This difference suggests that increasing current density above 2400 A/m^2^ reduces process energy efficiency. The results follow [18], where energy consumption increases with higher voltages and current densities.

**Figure 6:**
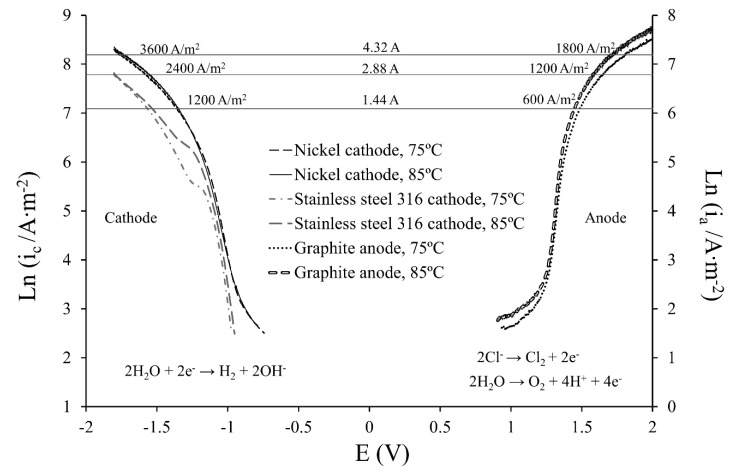
Kinetics of oxidation (Cl^−^/Cl_2_ and H_2_O/O_2_) and reduction kinetics (H_2_O/H_2_) using nickel, stainless steel 316 and graphite at 75 °C and 85 °C.

In this work, values of standard reduction potentials at different concentrations, temperatures and pH variations were determined, according to the Nernst equation. The standard reduction potential for cathodic and anodic reactions at 25 °C was −0.827 V and 1.358 V, respectively. At 75 °C and 85 °C, the equilibrium potential for the cathodic half-reaction resulted between −0.844 V and −0.870 V. On the other hand, anodic half-reaction values varied from 1.324 V to 1.336 V. The average difference between equilibrium potentials (∆E_e_) during experiments was between 2.17 V and 2.19 V. Variations of difference between equilibrium potentials can be attributed to concentration variation of electrolytes and pH changes during the electrodialysis process. Figure 6 was intended as a simplified Evans diagram showing experimental results and fitted curves for potentiodynamic sweeps. Cathodic reaction was characterized on nickel and sheet of stainless steel 316, and anodic reaction on graphite at 75 °C and 85 °C. Cathode and anode current densities (i_c_, i_a_) were determined by a quotient of current (I) and the corresponding effective area.

According to the results of potentiodynamic sweeps, it was determined that cathodic reactions were carried out under mixed control and mass transfer control when nickel and stainless steel 316 cathodes were used, respectively. The cathodic reaction on stainless steel 316 exhibited a lower exchange current density and a greater overpotential than the nickel one, therefore nickel as cathodic material offers performance with respect to cell voltage in the membrane electrodialysis cell. Futhermore, potentiodynamic tests in Figure 6 show that by using a nickel cathode, higher current densities could be reached. Regarding anodic reaction, a requirement to increase graphite anode area at least twice in order to reach cell current densities similar to that of nickel was observed. In this work, a graphite rod with an effective area of 24 cm^2^ was used as the anode, i.e., twice the area compared to the cathodes. Potentiodynamic sweeps results (Figure 6), suggest that for the same cathode and anode area, graphite would have a lower limiting current density than the current densities used. Experimentally, this was solved using a graphite bar with larger effective area, double that of the cathode area.

For the used current densities, no passivation process on the electrode surface was observed. In the potentiodynamic curves of Figure 6, there was not a sharp drop in current density in the range of the studied potentials, which would indicate a passive layer formation on electrode surface, reducing electric current passage [36].

Potential anolyte and catholyte drops were determined by the product of cell current and apparent electric resistance of the electrolyte ($R_{app}$) by Ohm’s law. Electrolyte electric resistance is related to Equation (5),

(5) $$R_{app}=\frac{1}{k}\frac{l}{A}$$

where *k* is the electrical conductivity, *A* is the cross-sectional area of electron flux and l is the distance between electrodes.

For current densities of 1200, 2400 and 3600 A/m^2^, average anolyte potential drops at 85°C were 0.24 V, 0.55 V and 0.71 V, respectively. For the same current densities and temperature, the average catholyte potential drops were 0.40 V, 0.79 V and 1.19 V, respectively.

From an energy point of view, using a current density of 1200 A/m^2^ obtained the lowest electrical consumption (see Table 3). However, this implies a slower production rate. It was determined that the process presented an acceptable specific electrical consumption up to a current density of 2400 A/m^2^. Results of this work indicate that working above 2400 A/m^2^ increases voltage losses (Experiment 3), however, it is believed that it is possible to decrease electrolytes potential drops (IR_a_ and IR_c_) to reduce cell voltage. This could be done by increasing the both recirculation flow rate and linear flow velocities [16], and decreasing electrodes and membrane separation [17].

Cell voltage values obtained in all experiments ranged from 3.86 to 7.82 V. Analysis of these results provide the basis to conclude that the cell voltage values obtained during this study show close similarity with the data available in the patent developed by Harrison and Blanchet [29], where cell voltage range was between 4.28 and 4.45 V, using an initial 21 wt% LiCl anolyte and an initial 2.4 wt% LiOH catholyte at 3000 A/m^2^. However, Buckley et al. [30] reported lower cell voltages (3.0–3.5 V) at current densities between 2000–3000 A/m^2^, using an initial 10–25 wt% LiCl anolyte and an initial 4–8 wt% LiOH catholyte at 2000–3000 A/m^2^. These variations can be attributed to current density, differences in cell design, and electrode types.

### 4.2. Temperature Effects

**Figure 7:**
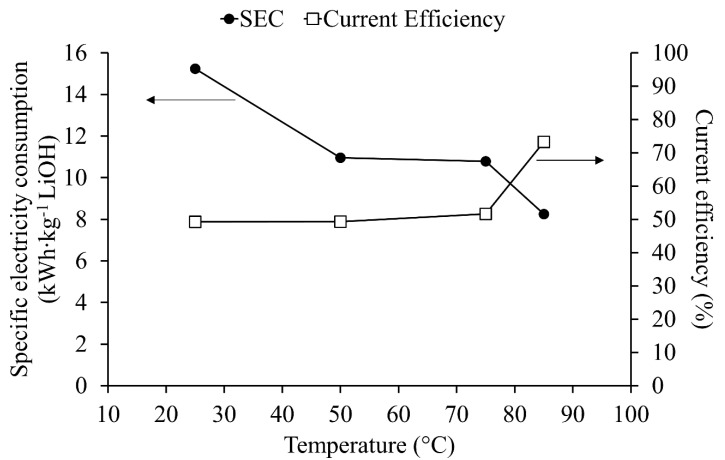
Specific electricity consumption and current efficiency variation with temperature at 2400 A∙m^−2^.

The effect of temperature can be observed when comparing experiments 2, 4, 9 and 10 (see Table 3). The results show temperature effects on LiOH production rate, electrical consumption in the membrane electrodialysis process, current efficiency and purity. Figure 7 shows temperature effects on specific electrical consumption and current efficiency at 2400 A/m^2^. Between 25°C and 50°C, a decrease of 28% in specific electrical consumption was achieved. This reduction can be attributed to the improvement of ionic mobility and conductivity in electrolytes with temperature increase. Specific electrical consumption (SEC) between 50°C and 75°C shows a slight decrease of 1.5%, and finally between 75 °C and 85 °C a decrease of 23% in SEC is measured. Temperature increase improves migration speed [17] and the diffusion rate of ions through the boundary layer next to the membrane surface and it can also affect membrane conductivity and volumetric expansion [37]. A slight variation in SEC between 50 °C and 75 °C was observed. For this, two explanations were proposed, first, a non-linear variation between temperature and conductivity of the electrolyte, and second, electrical resistance variations in cell components. Here, the cationic membrane may have more influence than bulk solution and boundary layers [38,39,40]. It is known that transport through membrane–electrolyte interface increases with temperature. Nevertheless, most reports in the literature have investigated this behavior between 10 °C to 40 °C, so more studies on this effect for temperatures above 50 °C are needed. Improvement at 85 °C could be attributed to changes in membrane stability [37] which allows better migration of counter-ions through the cationic membrane, at the cost of unwanted co-ions transport, reducing catholyte purity. However, further studies are necessary to establish this phenomenology. Current efficiency was 41% higher at 85 °C in comparison with lower temperatures.

**Figure 8:**
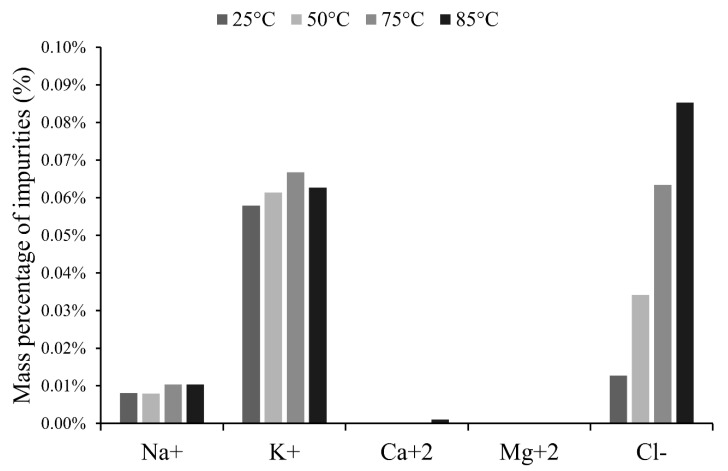
Mass of other ions in the final catholyte according to the temperature.

Temperature increase adversely affected product purity, facilitating the transport of impurities through the cationic exchange membrane. Figure 8 shows that temperature increase affected Cl^−^ co-ion leakage through the membrane. It is known that temperature also affects volumetric expansion of membrane [37], which could contribute to salt leakages. Furthermore, migration of other cations such as Na^+^ and K^+^ occurred but results do not clearly show a trend with temperature. 

With respect to energy parameters in the membrane electrodialysis cell, the best result was obtained at 85 °C. Related to product purity, it is clear that low temperature reduced salt leakages through the membrane.

### 4.3. Membrane Type Influence 

The effect of ion exchange membrane type on product purity can be observed when experiments 2 and 6 are compared. When using Nafion 115 and Nafion 117 membranes, purity percentages of 94.03% and 98.17% were obtained in lithium hydroxide monohydrate crystals, respectively.

Other results such as production rate, cell voltage, current efficiency and specific electrical consumption did not present significant differences according to the membrane type used.

In all results with both membranes, chemical analysis indicated the presence of other cations such as Na^+^, Ca^2+^ and K^+^ in the catholyte, which was attributed to migration of these ions through the membrane. On the other hand, migration of Mg^2+^ was not detectable and this can be explained by the low initial concentration of this cation. 

**Figure 9:**
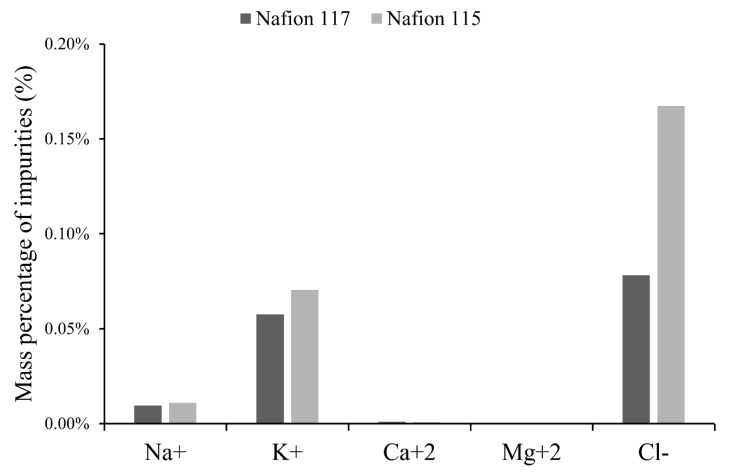
Mass of other ions in the catholyte according to type of membrane.

The Nafion 117 membrane is thicker and contains more monomer units than Nafion 115. A thicker membrane acts as a better containment barrier by decreasing the passage of unwanted elements. Chemical analysis results of the final catholyte indicated that the lowest transfer of Na^+^ and K^+^ ions was detected through the Nafion 117 membrane. For the Nafion 115 membrane, the amount of Na^+^ and K^+^ transported through the membrane was 21% and 29% higher than the Nafion 117, respectively. Contrarily, with the Nafion 115 a higher leakage of Cl^−^ in the catholyte was detected (Figure 9), suggesting Nafion 117 acts as a better containment barrier for salt leakage. Another probable cause of catholyte contamination with Cl^−^ is the dissolution of Cl_2_ gas present in air around the cell, which is generated by the anodic half-reaction. The Acrylic cell used was not a sealed model. Other studies indicate a high product purity could be obtained, depending on membranes types with higher permselectivity to lithium ions within a wide concentration range [27], allowing the attainment of a product that does not contain more than 0.5% of cations other than lithium and not more than about 0.05% of anions other than hydroxyl [28,30].

### 4.4. Cathode Material Influence 

The effect of the cathode material on energetic parameters (cell voltage, current efficiency and specific electrical consumption) can be observed by comparing experiments 4 and 5 (see Table 3). 

Use of nickel as a cathode has some advantages. It was observed that the current efficiency was 5% higher, the obtained production rate of LiOH was 23% higher, and the specific electrical consumption was reduced by 21%. 

**Figure 10:**
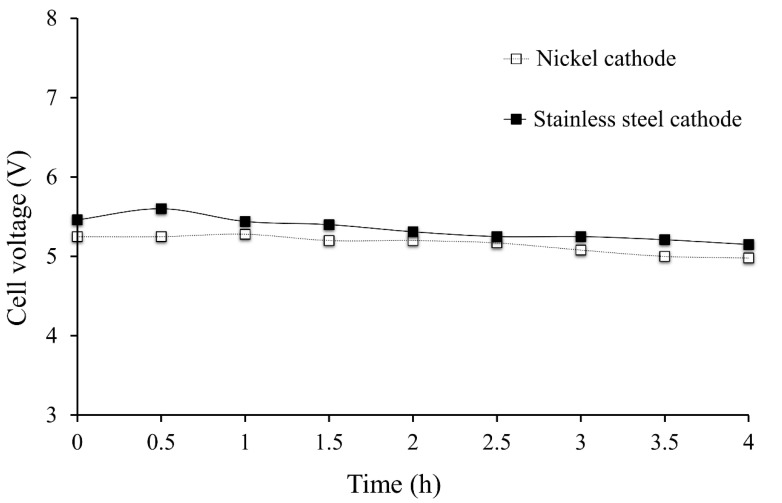
Cell voltage vs. time of operation by type of cathode at 2400 A/m^2^.

The best results obtained for nickel cathode can be attributed to the fact that this material has a lower electrical resistance compared to stainless steel 316. Moreover, there are effects of electrocatalysis related to different electrodes surfaces. This depends on material porosity and electrode effective area (related to the rugosity). An electrode is more electrocatalytic when an increase in its real surface area implies an overpotential reduction as the true current density is lowered. Nickel has advantages by being stable and electrically active. It is frequently used as the main component in the manufacture of cathodic materials for H_2_ evolution reactions. Catalytic activity is mainly due to electrode surface rugosity [41]. Cell voltage with respect to electrodes material is presented in Figure 10. During the experiments, lower cell voltage was obtained with a nickel electrode as cathode (3.45% lower than the stainless-steel electrode). From an energy point of view, the best results were obtained when using a nickel cathode.

### 4.5. Initial Concentration Influence 

The effect of the initial concentration can be observed when experiments 2, 8 and 11 are compared, corresponding to three different catholytes with initial concentrations of 1.15 wt% 2.30 wt% and 5.70 wt% of LiOH, respectively. The results indicate that initial concentration influenced specific electrical consumption and product purity. A high initial concentration in the catholyte implies a high electrolytic conductivity, therefore, a lower cell voltage can be expected. For 1.15 wt% and 2.30 wt% of LiOH cell voltage showed a variation within 4.89–5.01 V, while when LiOH 5.70 wt% was used, a 4.39 V cell voltage was obtained. High electrolytic conductivity implies a lower electrical resistance; therefore, a lower specific electrical consumption was obtained (8.89, 8.25 and 7.41 kWh/kg of LiOH was obtained for 1.15 wt%, 2.30 wt% and 5.70 wt% LiOH, respectively). 

**Figure 11:**
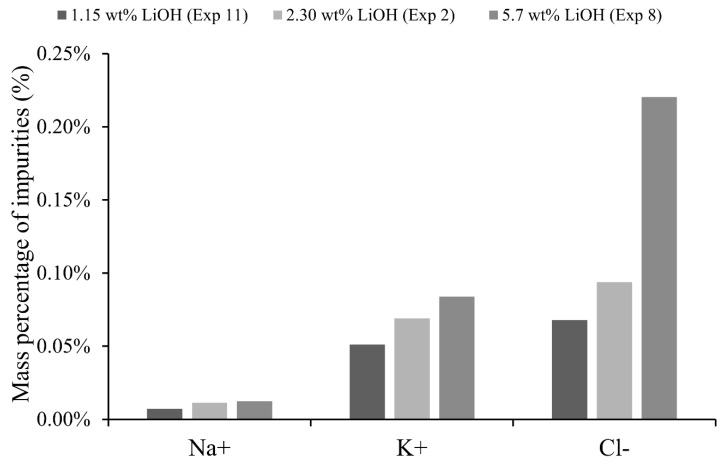
Impurities of Na, K and Cl in final catholyte according initial concentration of LiOH.

On the other hand, as can be observed in Figure 11, a high initial concentration in the catholyte adversely affects purity of the LiOH solution and therefore less product purity is obtained. This could be attributed to the fact that, for a high LiOH concentration of 5.7 wt%, counterion condensation on the membrane can occur, decreasing its permselectivity [26]. This could also be related to a greater chemical potential difference promoting other unwanted transport mechanisms. An example is that osmotic pressure difference between membrane and electrolyte could generate a water flux and pull some co-ions from the membrane to the LiOH solution. Another explanation is based on the Donnan dialysis of Li^+^, which due to high catholyte LiOH concentrations, would cause lithium transport to the anolyte and transport of other cations from anolyte to catholyte. However, during the whole operation time, Li^+^ concentration in the anolyte was higher than that in the catholyte, so the concentration difference indicates that transport by Donnan dialysis would not occur under the studied conditions. It is interesting to compare experiment 1 and experiment 12, where anolyte 1-catholyte 2 (2.30 wt% LiOH) and anolyte 2-catholyte 1 (1.15 wt% LiOH) were used, respectively. At the same current density, experiment 12 with a higher concentration difference of anolyte and catholyte shows a 4.2% lower cell voltage, 3.3% lower specific electrical consumption and a 2.5% lower production rate of LiOH. However, current efficiency was 15% higher for experiment 1. A high concentrated anolyte can cause a greater concentration of co-ions in the membrane according to Donnan exclusion. This can decrease efficiency and permselectivity of the membrane. For experiment 12, chemical analysis showed a 105% higher Cl^−^ concentration in final catholyte compared to experiment 1.

Regarding production rate, slight differences were observed according to initial catholyte and anolyte concentrations. This suggests that lithium transport rate depends mainly on electric current (Equation (1)), as long as there is sufficient availability of Li^+^ ions for migration near the surface of the membrane.

From a product purity point of view, it is better to use a low initial catholyte concentration and an anolyte with initial concentration lower than LiCl 32 wt%.

### 4.6. Final Product Chemical Characterization

The resulting catholyte after each experiment was subjected to evaporation, crystallization and drying to obtain solid LiOH·H_2_O crystals. Subsequently, the crystals were washed and dried again in a CO_2_ free atmosphere. Chemical analysis results for the obtained crystals are presented in Table 4. Impurity migration across the membrane influences the purity achieved in each experiment. The best results were obtained for experiment 4, when current density was 2400 A/m^2^, temperature was 75 °C, the membrane was Nafion 117 and the cathode was nickel. These conditions allowed the attainment of high purity LiOH solutions with specific electrical consumption in the electrodialysis membrane process of 10.79 kWh/kg LiOH.

**Figure 12:**
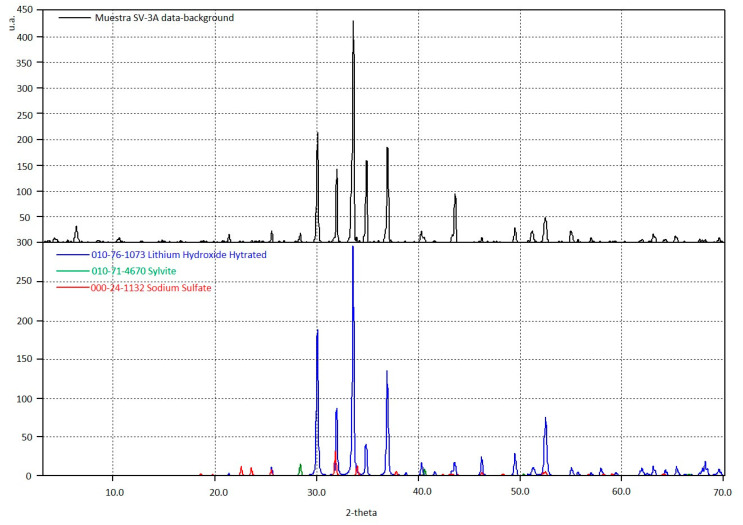
Diffraction pattern of the crystals obtained in the experiment 2.

Based on performing X-ray diffraction analysis, the presence of LiOH∙H_2_O was verified. The diffractogram of Figure 12 corresponds to experiment 2.

### 4.7. Product Purity and Specific Electricity Consumption 

During experiments, the influence of different variables on energy efficiency and product quality was observed.

Results indicate that product purity was between 93.65 and 99.93%, while specific electrical consumption ranged from 7.25 to 15.24 kWh/kg LiOH at cell current densities between 1200 and 3600 A/m^2^. In the literature, specific electrical consumption varies between 5 to 15 kWh/kg LiOH at different operating conditions [29], obtaining at best a result of 5 kWh/kg LiOH from a similar initial catholyte concentration (approximately 2.4 wt% LiOH). Our specific electrical consumption results were similar to those previously reported. In relation to the amount of impurities obtained in other studies, Brown [28] indicates obtaining a product not containing more than 0.5% of cations other than lithium and not more than about 0.05% anions other than hydroxyl. In the present work, the percentage of other cations was between 0.05% and 0.11% and the lowest presence of Cl^−^ in catholyte was 0.01 wt% in experiment 9 at 25 °C.

In this work, it is determined that current density and cathode type have an impact on electrical consumption. In a similar way, it was observed that membrane thickness impacts product purity. On the other hand, there were two variables influencing both specific electrical consumption and product purity. These are electrolyte temperature and initial catholyte concentration. In order to ensure a high purity of the product with a low electricity consumption, the optimum conditions must be found.

It is possible that working at temperatures above 80 °C causes a decrease in the membrane’s ion exchange capacity and promotes structural changes thereof. In fact, several previous studies have demonstrated that temperature significantly impacts upon NF membrane performance. According to Goosen et al. [42], the polymeric membrane is sensitive to changes in feed temperature. They reported an increase of up to 60% in permeate flux when the feed temperature was increased from 20°C to 40 °C. A linear relation between temperature and water flux by NF performances has been reported [43]. It is explained that flux increase with temperature is attributed to membrane material thermal expansion. In a study on the effect of temperature on permeation characteristics of NF membranes, Sharma et al. [44] suggested that with increasing temperature, the average pore size increases and pore density decreases because thermal expansion of the polymer constitutes the active layer on thin-film composite membranes. This could be the cause of reduction in rejection of organic solutes by NF membranes with increasing temperature.

An optimum current density allows a high rate of LiOH generation at low energy cost. If current density is low, ion migration is controlled and has low specific electrical consumption. However, LiOH generation rate is slow and may not meet the desired production requirements on an industrial scale. For operating conditions in this work, it was determined that for high energy efficiency and acceptable production rate, the current density should not exceed 2400 A/m^2^. 

Electrolyte temperature and concentration impact on product purity and specific electrical consumption were studied. Changes in any of these variables simultaneously cause increased electrical consumption and higher purity, or vice versa. It was found that working at low temperatures reduced Cl^−^ leakage to catholyte, however, electrolytic conductivity was lower and higher specific electrical consumption was obtained. Heating the electrolyte would help reduce electrical consumption at the cost of heat consumption increase.

The highest purity was obtained at a 75°C temperature, however, high electrical consumption was obtained (11.65–13.73 kWh/kg LiOH). For the production of battery grade LiOH∙H_2_O as raw material for cathode materials, the priority is high purity. 

Cation transport other than lithium through membrane and their presence in the final product depends mainly on initial electrolyte concentration and temperature. These indicate the importance of a proper pre-treatment for impurity removal from natural brines. 

It must be considered that, in this work, the effect of recirculation flow velocity was not analyzed due to used cell limitations, so finding optimal flow may provide a better scenario with respect to energy consumption. Optimizing and analyzing cell design is the next step in the development effort for this research. A high flow rate would be expected to reduce specific electrical consumption by improving ion transport in the electrolyte and membrane surface boundary layer. This aspect shall be supported with the use of suitable cationic membranes, with optimized ion-exchange capacity, mechanical stability and water uptake that are not affected by loss of permselectivity at high salt concentrations and in the presence of bases. In addition, the use of selective monopolar membranes would contribute the attainment of high purity LiOH in the presence of other divalent cations such as Ca^2+^ and Mg^2+^.

### 4.8. Further Work

With the results at this stage, the construction of a new bench scale cell is to be defined, including: (a) a closed reactor design, allowing an electrolyte recirculation flow increase, containing devices to capture gases generated in each compartment, and a shorter distance between electrodes; (b) study of new electrocatalytic materials such as electrodes and their effect on electrochemical kinetics, such as RuO_2_/Ti, IrO_2_/Ti, Pt/Ti, and study of current distribution lines according to their geometry; (c) development of a predictive mathematical model of LiOH production using this technology, coupling electrochemical kinetics and transport mechanisms across the membrane (d) study of the effect of higher impurity concentrations in the electrolyte such as Mg^2+^ and Ca^2+^ on the membrane.

## 5. Conclusions

In all experiments, the obtained LiOH∙H_2_O presents a purity between 93.65 and 99.93%, the highest purity and battery grade were achieved in experiment 4. This was achieved with membrane Nafion 117, nickel as the cathode material, at a temperature of 75 °C and 2400 A/m^2^ current density. The results indicated that product purity was favored by temperatures below 75 °C, with a thicker membrane (Nafion 117) and low initial electrolyte concentration.

From the point of view of energy efficiency in the membrane electrodialysis cell, the lowest specific electrical consumption (7.25 kWh/kg LiOH) was obtained with a 1200 A/m^2^ current density, the temperature was 85 °C, nickel was used as the cathode and with an initial catholyte concentration of 5.70 wt% LiOH. However, using a low current density presented the disadvantage of decreasing LiOH production. The average specific electrical consumption (SEC) was 9.9 kWh/kg LiOH.

At temperatures between 25 °C and 75 °C current efficiencies in the range of 49.0–51.7% were observed. It is necessary to improve the process to reduce specific electrical consumption and simultaneously achieve better product purity. This improvement can be achieved by developing more selective membranes and finding optimal flow rate and the minimum electrode distance.

The work carried out demonstrates the feasibility of using a membrane electrodialysis process to obtain high purity LiOH·H_2_O (battery grade) and provides information on process sensitivity to variations on different operating conditions. 

## Figures and Tables

**Table 1:** Anolytes chemical composition.

Species	Anolyte 1 (gr/100 gr Solution)	Anolyte 2 (gr/100 gr Solution)
Li^+^	2.200	4.152
Na^+^	0.038	0.071
K^+^	0.200	0.378
Ca^2+^	0.003	0.007
Mg^2+^	0.0002	0.0003
Cl^−^	11.481	21.660
SO_4_^2−^	0.008	0.017

**Table 2:** Operating conditions for each experiment.

N° Experiment	1	2	3	4	5	6	7	8	9	10	11	12
Current density (A∙m^−2^)	1200	2400	3600	2400	2400	2400	2400	2400	2400	2400	2400	1200
Cathode material	Nickel	Nickel	Nickel	Nickel	Stainless Steel 316	Nickel	Stainless Steel 316	Nickel	Nickel	Nickel	Nickel	Nickel
Initial Anolyte ^1^	Anolyte 1	Anolyte 1	Anolyte 1	Anolyte 1	Anolyte 1	Anolyte 1	Anolyte 1	Anolyte 1	Anolyte 1	Anolyte 1	Anolyte 1	Anolyte 2
Initial concentration of LiOH in the catholyte (wt %)	2.30	2.30	2.30	2.30	2.30	2.30	2.30	5.70	2.30	2.30	1.15	1.15
Temperature (°C)	85	85	85	75	75	85	85	85	25	50	85	85
Membrane (NAFION)	117	117	117	117	117	115	115	117	117	117	117	117

^1^ See Table 1.

**Table 3:** Results obtained in each experiment with membrane electrodialysis cell.

N° Experiment	1	2	3	4	5	6	7	8	9	10	11	12
Current density (A∙m^−2^)	1200	2400	3600	2400	2400	2400	2400	2400	2400	2400	2400	1200
Cathode material	Nickel	Nickel	Nickel	Nickel	Stainless Steel 316	Nickel	Stainless Steel 316	Nickel	Nickel	Nickel	Nickel	Nickel
Initial Anolyte	Anolyte 1	Anolyte 1	Anolyte 1	Anolyte 1	Anolyte 1	Anolyte 1	Anolyte 1	Anolyte 1	Anolyte 1	Anolyte 1	Anolyte 1	Anolyte 2
Initial concentration of LiOH in the catholyte (wt %)	2.30	2.30	2.30	2.30	2.30	2.30	2.30	5.70	2.30	2.30	1.15	1.15
Temperature (°C)	85	85	85	75	75	85	85	85	25	50	85	85
Membrane (NAFION)	117	117	117	117	117	115	115	117	117	117	117	117
Production rate of LiOH (mol∙m^−2^∙h^−1^)	27.9	61.3	102.1	47.9	39.0	61.2	49.7	59.4	44.1	44.1	54.5	27.2
Average cell voltage (V)	4.03	5.01	7.82	5.15	5.34	5.06	4.66	4.39	6.70	4.83	4.89	3.86
Current efficiency (%)	70.00	73.27	76.69	51.65	48.99	73.58	52.00	72.13	49.22	49.32	60.83	60.71
SEC (kWh/kg LiOH)	7.25	8.25	11.65	10.79	13.73	8.27	9.41	7.41	15.24	10.96	8.89	7.01

**Table 4:** Solid product chemical analysis for all experiments, in wt%.

	Experiment											
	1	2	3	4	5	6	7	8	9	10	11	12
LiOH·H_2_O Before wash and dry	93.32	94.93	92.81	97.01	94.77	90.22	93.01	90.74	97.31	97.02	94.91	93.63
LiOH·H_2_O After wash and dry with 3% humidity	96.37	98.17	95.17	99.93	99.44	94.03	95.83	93.65	99.22	98.12	95.21	98.92
